# Combination of CEACAM5, EpCAM and CK19 gene expressions in mediastinal lymph node micrometastasis is a prognostic factor for non-small cell lung cancer

**DOI:** 10.1186/s13019-023-02297-z

**Published:** 2023-06-13

**Authors:** Hande Süer, Suat Erus, Ekin E. Cesur, Ömer Yavuz, Orhan Ağcaoğlu, Pınar Bulutay, Tamer T. Önder, Serhan Tanju, Şükrü Dilege

**Affiliations:** 1 Graduate School of Health Sciences, Acıbadem Mehmet Ali Aydınlar University, Istanbul, Turkey; 2 Thoracic Surgery Department, Koç University School of Medicine, Istanbul, Turkey; 3 Thoracic Surgery Department, Vehbi Koç Foundation American Hospital, Istanbul, Turkey; 4 General Surgery Department, Koç University School of Medicine, Istanbul, Turkey; 5 Pathology Department, Koç University School of Medicine, Istanbul, Turkey; 6 Koç University School of Medicine, Istanbul, Turkey

**Keywords:** Cancer, NSCLC, Micrometastasis, Mediastinal lymph node micrometastasis, Skip metastasis

## Abstract

**Background:**

Lung cancer is known as the most common and highly metastatic form of cancer worldwide. Tumour node metastasis (TNM) staging is the gold standard classification system for the decision-making process for appropriate treatment. Particularly N status has the most important prognostic value in the absence of distant metastasis. Traditional diagnostic methods are capable of detecting metastasis; however, they may fail to detect micrometastasis, which plays a role in disease recurrence and patients' long-term survival. Occult micrometastasis can change the tumour's TNM staging and, consequently, the patient's treatment regimen.

**Methods:**

The median number of three lymph node tissues were collected from 30 patients who underwent surgery for non-small cell lung cancer. Lymph node tissues were collected from different lymph node stations according to the location of the patient's tumour. CK19, EpCAM and CEACAM5 gene expressions were analysed in tissues using quantitative real-time polymerase chain reaction to detect micrometastasis in distant lymph nodes.

**Results:**

Triple positivity was seen in 26 out of 30 patients which 19 patients were upstaged from N0 to N2. While survival was not significantly affected between upstaged and non-upstaged patients, patients upstaged with multiple-station N2 had a significantly higher recurrence and lower survival compared to single-station N2.

**Conclusion:**

A combination of CK19, EpCAM and CEACAM5 gene expressions in lymph nodes can be used to identify micrometastasis which postoperatively may be used as a tool to predict patients’ recurrence and survival.

**Supplementary Information:**

The online version contains supplementary material available at 10.1186/s13019-023-02297-z.

## Introduction

Primary lung cancer is one of the deadliest diseases, which has caused approximately 1.7 million deaths per year worldwide [1]. **T**umour **N**ode **M**etastasis (TNM) staging system is commonly used to classify the extent of spread in patients with lung cancer, and it is crucial to decide on the appropriate treatment. In the absence of distant metastasis mediastinal node (MLN) involvement, the N factor is the most important prognostic factor in NSCLC as preoperatively it is strategic to assess a proper treatment. For example, a neoadjuvant therapy or exclude surgery may be required in N2 status, while N1 status should guide the decision of upfront surgical resection [2]. Unfortunately, accurately assessing nodal status preoperatively is still challenging due to the lack of sensitivity and specificity of current imaging techniques and reduced use of invasive staging procedures such as mediastinoscopy and endobronchial ultrasound-transbronchial needle aspiration [2]. Therefore, accurate staging of a patient usually happens postoperatively however, occult micrometastasis can still be missed by the existing detection techniques.

Occult micrometastasis in lymph nodes is defined as a group of tumour cells measuring between 0.2 and 2 mm, which cannot be detected by standard diagnostic tests preoperatively and only detected after pathologic investigation of the surgical specimen. It is not uncommon as its prevalence was reported to be around 20% in clinically lymph node-negative patients undergoing surgical resection [3]. The presence of micrometastasis is found to be associated with disease recurrence and decreased long-term survival rates in non-small cell lung cancer (NSCLC) patients [4, 5]. Due to its effect on recurrence and prognosis, detecting occult micrometastasis is critical for staging.

There are several techniques used to detect micrometastasis. Conventional histopathological methods such as haematoxylin and eosin (H&E) staining or immunohistochemistry (IHC) using specific markers could detect micrometastasis at a certain sensitivity and specificity [6] and molecular detection methods that are gel-based qualitative polymerase chain reaction (PCR) and quantitative reverse transcriptase PCR (RT-PCR) have been used for more rapid detection. IHC is one of the standard histopathological methods to detect MLN micrometastasis. It is a qualitative method that selectively recognises proteins in the cells of tissue sections by using specific antibodies that bind to the protein of interest. Although IHC is superior to H&E, it still has constraining factors for its clinical use, such as the increased cost and labour and antibody dependency, as the sensitivity and selectivity of the test will always be as high as the affinity of the antibody to its target protein. In a typical lung cancer resection, approximately 10–20 lymph nodes are removed. To detect micrometastasis, multiple sections need to be taken from each lymph node and analysed by a pathologist, which is almost impossible to be applied to every patient as a routine examination [7]. RT-PCR is a widely used and objective molecular detection method that can quantify the expression of a particular gene, even though it is scarcely expressed in samples [8]. RT-PCR uses automated processing and analysis, allows many samples to be tested simultaneously, and the entire procedure can be completed within a day. These reduce the subjectivity of the analysis and make it more suitable for clinical application due to the reduction of manual labour [7]. RT-PCR is applied to the detection of micrometastasis in lymph nodes with the principle of using specific genes that are exclusively expressed in tumour cells but not in HLNs. Therefore, identifying the genes that are strictly associated with the type of cancer is essential for the specificity and sensitivity of the diagnosis. Studies comparing IHC and RT-PCR on the same samples subsequently showed that a well-designed and controlled RT-PCR is a more sensitive, standardised, and objective approach to detecting micrometastasis [7, 9]. In the meta-analysis of Jeong et al. [10], different detection methods are evaluated through subgroup analysis and stated that the PCR method had a higher detection rate compared to IHC. Yet, there was no significant change in the survival rate between cases with nodal micrometastasis and without nodal micrometastasis of N1 and N2 nodes in the PCR method; thus, the impact of a higher detection rate on survival still needs to be evaluated.

To enhance the sensitivity of RT-PCR, combining markers would be advantageous such as using epithelial and tumour-specific markers together, as the use of a single marker may lead to false-negative results due to tumour heterogeneity [11]. Since NSCLC may present heterogeneous gene expression patterns, selecting appropriate and relatively common markers is essential. There have been many biomarkers used to detect micrometastasis in MLNs: surfactant proteins that are specifically expressed in lung epithelium and NSCLC [7, 12], CK19 and cytokeratin 7, which are epithelial markers that form intermediate filaments [7, 11], mucin-associated protein (MUC1) which is a cell surface glycoprotein expressed in lung tissues [11], EpCAM that is considered as a stand-alone marker for NSCLC [7, 13, 14] and carcinoembryonic antigen (CEA) which is a cell adhesion molecule and widely used tumour marker that is expressed in a majority of carcinogeneses such as gastric, breast, colorectal and NSCLC [15–19]. All these markers are known to be absent in lymphatic tissues or haematopoietic cells.

To evaluate MLN micrometastases in patients who undergo surgical resection for NSCLC [16, 20]., we used a combination of markers, which are cytokeratin 19 (CK19) and epithelial cell adhesion molecule (EpCAM), that are both known as stand-alone markers for NSCLC and expressed regardless of histologic subtype of NSCLC [7, 11], and CEACAM5, which is a commonly used tumour marker. While these markers are expressed on epithelial tumour cells their expressions are scarce in lymph nodes. Additionally, we investigated whether detecting occult micrometastasis in MLNs would have any impact on patients’ survival as macrometastasis and postoperative detection of micrometastasis through this combination of biomarkers could hold a prognostic value.

## Material and methods

### Patients

From July 2013 through July 2014, 32 consecutive patients with operable NSCLC, according to NCCN guidelines stage 2B and/or lower, and with no prior neoadjuvant treatment regimens were enrolled in the study. Before the operation, every patient underwent a positron emission tomography (PET) scan and brain magnetic resonance imaging (MRI) to confirm that there was no distant metastasis. Invasive staging techniques such as bronchoscopy and mediastinoscopy were only applied to patients whose PET results are ambiguous for a mediastinal disease.

The tumour stage was classified according to the 8th edition lung cancer staging system by the International Association for the Study of Lung Cancer [21]. Patients who had MLN metastasis detected after the post-operational pathological examinations were excluded from the study. All patients were operated on at the Department of Thoracic Surgery, Koç University Hospital, Istanbul, Turkey, and had given written informed consent. After the primary surgery, patients were examined every six months until July 2019 when the last recruited patient would fulfil a 5-year follow-up period. The examination included a chest computed tomography in routine and a PET scan when necessary.

### Samples

TNM staging systems were applied for histopathological classification, grading, and staging of the tumours. ESTS guidelines were followed for a complete resection of NSCLC, and according to the tumours' location (right or left or upper or lower), nodal dissection was performed. The lobar location of tumours and the lymphatic drainages of the corresponding lobes were the determining factors in the removal of the lymph nodes. Following the resection, the lymph node samples were immediately evaluated by a pathologist for macrometastasis, and lymph node tissue samples found negative for macrometastasis were collected in RNAlater (Qiagen) and stored at − 80 °C until the analysis. The median number of tissues obtained from different lymph node stations for each patient was three.

To determine heatmap's lower and upper gene expression limits, healthy lymph nodes (HLN) (n = 6), which were collected from cancer-free patients who underwent lobectomy due to bronchiectasis, and metastatic lymph nodes (MetLN) (n = 3) that were collected from NSCLC patients whose lymph nodes confirmed to have macrometastasis by a pathologist.

After RNA isolation, the remaining lymph nodes of the patients were sent to the pathology laboratory for a detailed pathological examination. Then these lymph nodes were cut with serial sections in the pathology department and re-evaluated for micrometastatic foci. They were also stained with CK19 IHC stain simultaneously.

### Total RNA extraction and cDNA synthesis

Total RNA was extracted from preserved samples with a Quick-RNA Miniprep kit (Zymo Research) according to the manufacturer's instructions following a homogenisation and Proteinase K treatment. The quality and quantity of the RNA were measured by the NanoDrop spectrophotometer (Thermo Scientific). cDNA was reverse transcribed from 1 μg of total RNA in a final 100 μl reaction mixture with random hexamer priming (Invitrogen) and Superscript III Reverse Transcriptase (Invitrogen).

Primers for EpCAM, CEACAM5 and CK19 were purchased from Sentromer (Istanbul, Turkey). Primer sequences are listed in Table 1.

**Table 1:** Properties of primers used in RT-PCR

Genes	Primers	Melting temperature (Tm)	Amplification efficiency
*EpCAM*		60 °C	1 (R^2^ = 0.98)
Forward	5′- TGATCCTGACTGCGATGAGAG-3′		
Reverse	5′- CTTGTCTGTTCTTCTGACCCC-3′		
*CEACAM5*		60 °C	0.99 (R^2^ = 0.97)
Forward	5′-TCTTGGCTGATTGATGGGAAC-3′		
Reverse	5′-CACTGGCTGAGTTATTGGCCT-3′		
*CK19*		55 °C	0.99 (R^2^ = 0.93)
Forward	5′-AACGCCGAGCTAGAGGTGA-3′		
Reverse	5′-GGATGGTCGTGTAGTAGTGCC-3′		
*GAPDH*		55–60 °C	1 (R^2^ = 0.99)
Forward	5′-AGGGCTGCTTTTAACTCTGGT-3′		
Reverse	5′-CCCCACTTGATTTTGGAGGGA-3′		

RT-PCR was carried out in a LightCycler^®^ 480 II Instrument (Roche Diagnostics). RT-PCR assays to measure the relative amount of EpCAM, CEACAM5 and CK19 mRNA was performed using a total volume of 20 μl reaction mix containing 10 μl LightCycler^®^ 480 SYBR Green I Master Mix (Roche), 2 μl 2.5 mM primer set, 2 μl cDNA (1000 ng/100 μl) and 6 μl nuclease-free water (Thermo Scientific). RT-PCR cycles were pre-incubation at 95 °C for 5 min, followed by 40 cycles of 95 °C for 10 s and either 61 °C or 55 °C for 30 s and 72 °C for 30 s. PCR products were also analysed by melting curves to confirm the specificity of the primers under reaction conditions. All melting curves revealed well-defined peaks with the expected melting temperatures. Controls that contain no cDNA were included in every run to monitor potential contamination. The threshold cycle number (Ct) represents the cycle number in which the amount of amplified target product reached a certain threshold.

All primers were designed to amplify the genes of interest and were optimised using cDNA isolated from RNA of cancer-free lung tissue. Linear regression was plotted using 1:1 serial dilution of the cDNA starting from 40 ng per 20 μl reaction mixture. Amplification efficiency (AE) was calculated according to the following equation [11]:

$$Amplification\;Efficiency\left( {AE} \right) = 1 - 10^{ - 1/m}$$

where m represents the slope of the line determined by the serial dilution.

The amount of cDNA was normalised to internal control GAPDH levels and relative to mRNA expression in cancer-free lung tissue as a standard. Relative quantity was calculated by the following equation [11]:

$$Relative\;Quantity \left( {RQ} \right) = \left( {1 + AE} \right)^{ - \Delta \Delta Ct}$$

where,

$$\begin{aligned} \Delta \Delta Ct = & \left[ {\left( {Ct_{gene\;of\;interest} - Ct_{internal\;control} } \right)_{sample} } \right. \\ & \left. { - \left( {Ct_{gene\;of\;interest} - Ct_{internal\;control} } \right)_{standard} } \right] \\ \end{aligned}$$

Expression levels of EpCAM, CEACAM5 and CK19 were analysed in HLNs and MetLNs to determine the minimum and maximum expression levels, respectively. A threshold of minimum expression level was determined by the upper 99% CI of the mean relative quantity value for these three genes. Lymph nodes were considered positive for micrometastases when all duplicate assays crossed the threshold values for the three markers' expressions.

### Statistical analyses

The descriptive statistics for clinical data were expressed as mean ± SD. The Pearson chi-square test or Fisher exact test was performed to measure any possible correlations among micrometastatic patients and the patients' clinicopathologic characteristics. The Kaplan–Meier method and the Log-rank test were used to analyse the survival time between micrometastatic and non-micrometastatic patients. For the patients who were still alive, data were censored at the time of the last follow-up visit. The minimum level of significance was set at *p* < 0.05. The statistical analysis was carried out using the SPSS 22.0 software.

## Results

Each of the 32 patients underwent complete resection for NSCLC. Two patients were excluded from the study after postoperative pathological examinations due to N2 disease. Patients who continued with the study included 15 males and 15 females. Histological types of cancer were predominantly adenocarcinoma (73.3%), followed by squamous cell carcinoma (20%) and large cell carcinoma (6.7%). The pathological stage was diagnosed as stage I in 18 patients, stage II in 7 and stage III and IV in 4 and 1 patients, respectively (Table 2). Patients were followed up for five years after the last patient was recruited. The median follow-up was 70 months.

**Table 2:** Demographics of the patient cohort (n = 30)

Parameters	Patients n (%)
*Age (year)*	
< 65	15 (50)
> 65	15 (50)
*Sex*	
Men	15 (50)
Female	15 (50)
*Histology*	
Squamous cell carcinoma	6 (20)
Adenocarcinoma	22 (73.3)
Large cell carcinoma	2 (6.7)
*pTNM stage*	
I	18 (60)
II	7 (23.3)
III	4 (13.3)
IV	1 (3.3)
*Nodal status*	
N0	24 (80)
N1	8 (20)
*Recurrence*	
Absent	23 (76.7)
Present	7 (23.3)

First, to determine lower and upper gene expression thresholds, EpCAM, CEACAM5 and CK19 expression levels were analysed in HLN and MetLNs. In this study, MetLNs were used to determine the upper limit of the heatmap as MetLNs were found to have higher expression of these three genes than tumour foci (data not shown). However, the combination of gene expressions in MetLNs was representative of the upper limit and only used for comparative purposes. Since we aimed to identify genes, whose expressions were scarce in HLNs, we focused mainly on the lower threshold. The mean expression level of EpCAM, CEACAM5 and CK19 in HLNs were 0.023 ± 0.011, 0.073 ± 0.141 and 0.032 ± 0.025, respectively. The samples were considered positive for EpCAM, CEACAM5 and CK19 if the relative quantity value was greater than 0.042, 0.305 and 0.074, respectively, corresponding to the upper 99% CI of the mean values in control samples (Table 3).

**Table 3:** Relative quantity values of EpCAM, CEACAM5 and CK19 in HLNs

Healthy lymph nodes (n = 6)	Relative quantity of		
	EpCAM	CEACAM5	CK19
Minimum	0.007	0.001	0.000
25% Percentile	0.011	0.001	0.010
Median	0.024	0.006	0.031
75% Percentile	0.033	0.145	0.051
Maximum	0.038	0.355	0.074
Mean	0.023	0.074	0.032
Std. deviation	0.011	0.141	0.025
Std. error of mean	0.005	0.057	0.010
Lower 99% CI of mean	0.004	− 0.158	− 0.010
Upper 99% CI of mean	0.042	0.305	0.074

The upper 99% CI of the mean is accepted as the minimum RQ level of genes in patients' samples

**Fig. 1:**
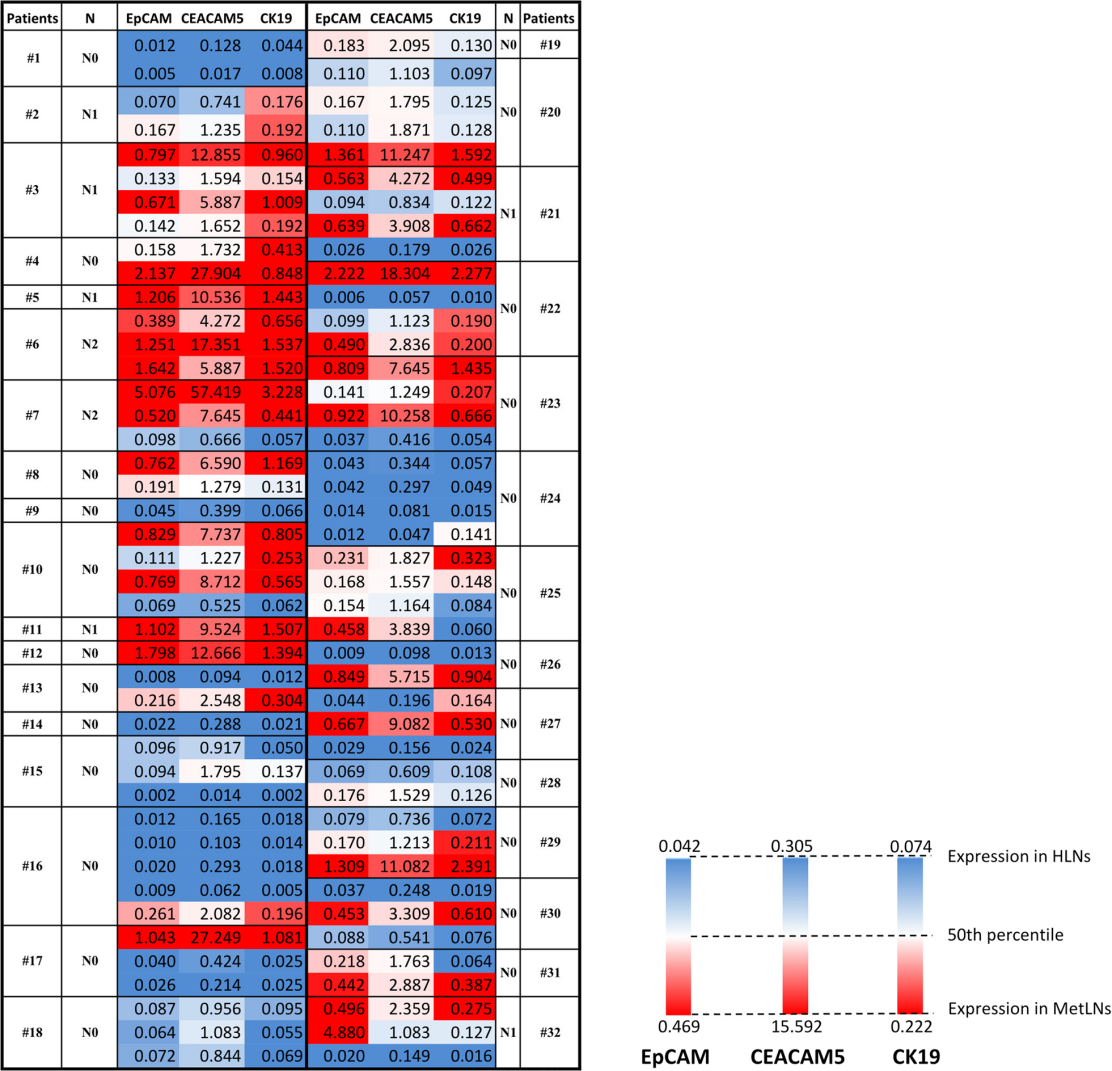
Heatmap of relative quantity values for EpCAM, CEACAM5 and CK19 in patient samples

Once the thresholds were determined for three genes, 80 lymph nodes were analysed in 30 patients. EpCAM, CEACAM5 and CK19 mRNA detection in histologically tumour-free MLNs revealed that 60 out of 80 lymph nodes were positive for EpCAM, 60 for CEACAM5, and 51 for CK19, whereas 49 (61.25%) were found positive for all three genes (Fig. 1). We considered the lymph nodes micrometastatic, where all three markers were positive. Micrometastasis is detected in 49 of 80 lymph nodes (61.25%) and consequently, 26 of 30 patients (86.67%) were considered positive for micrometastases. Considering patients’ tumour location and micrometastasis presence in lymph node stations, we revealed that one patient was upstaged from N0 to N1, six patients were upstaged from N1 to N2, 19 patients from N0 to N2 and no micrometastasis detected in four patients.

**Fig. 2:**
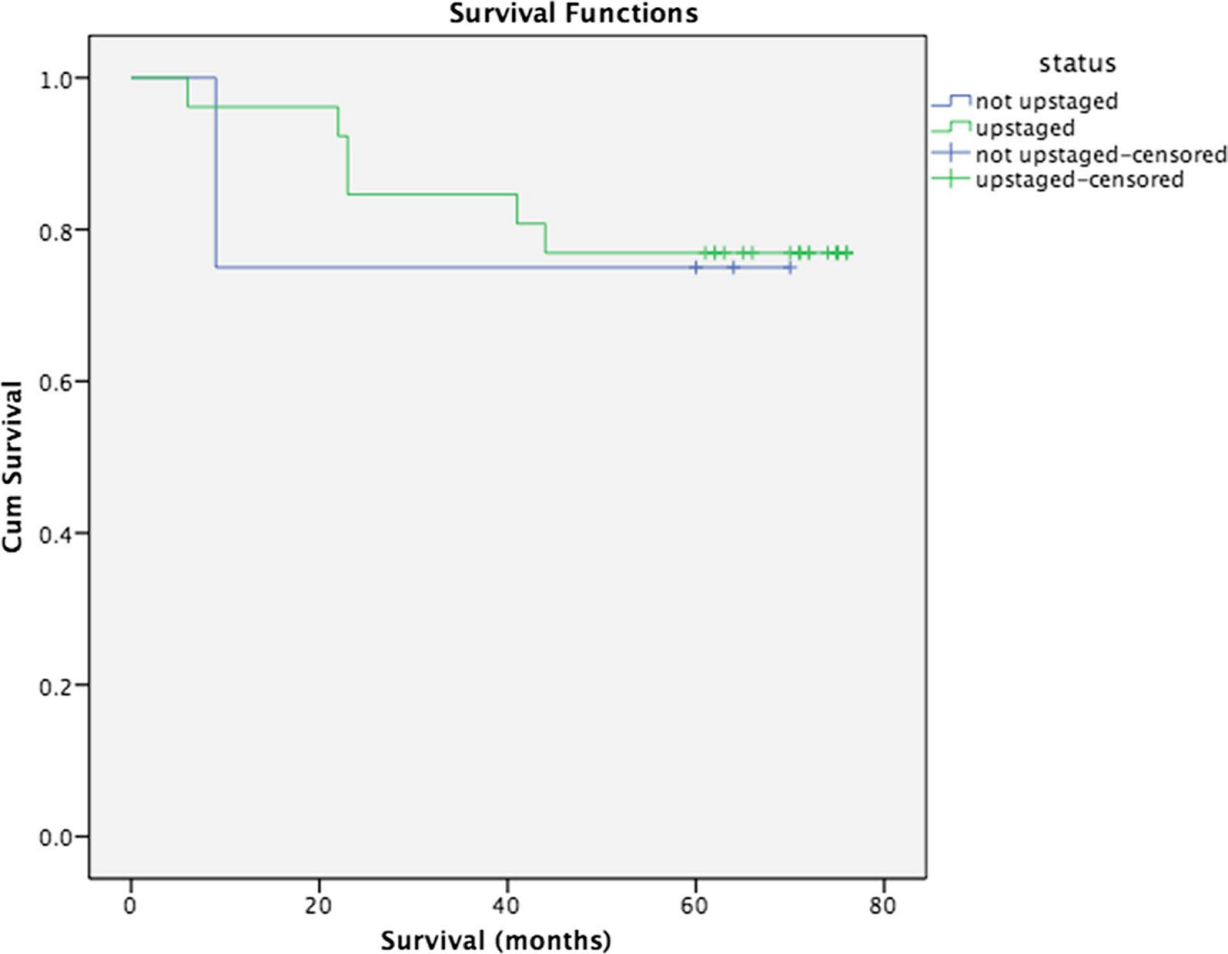
Survival analysis of upstaged patients after a 5-year follow-up period

First, we would like to examine if the presence of micrometastasis in MLNs would have any impact on patients’ survival and the recurrence of the disease. To find out we performed Kaplan–Meier survival analyses. It is observed that the patients who were upstaged tended to have slightly poorer survival compared to non-micrometastatic patients, but without a statistical significance (median survival of 62 months for upstaged vs 79 months for not upstaged, *p*: 0.88) (Fig. 2).

**Fig. 3:**
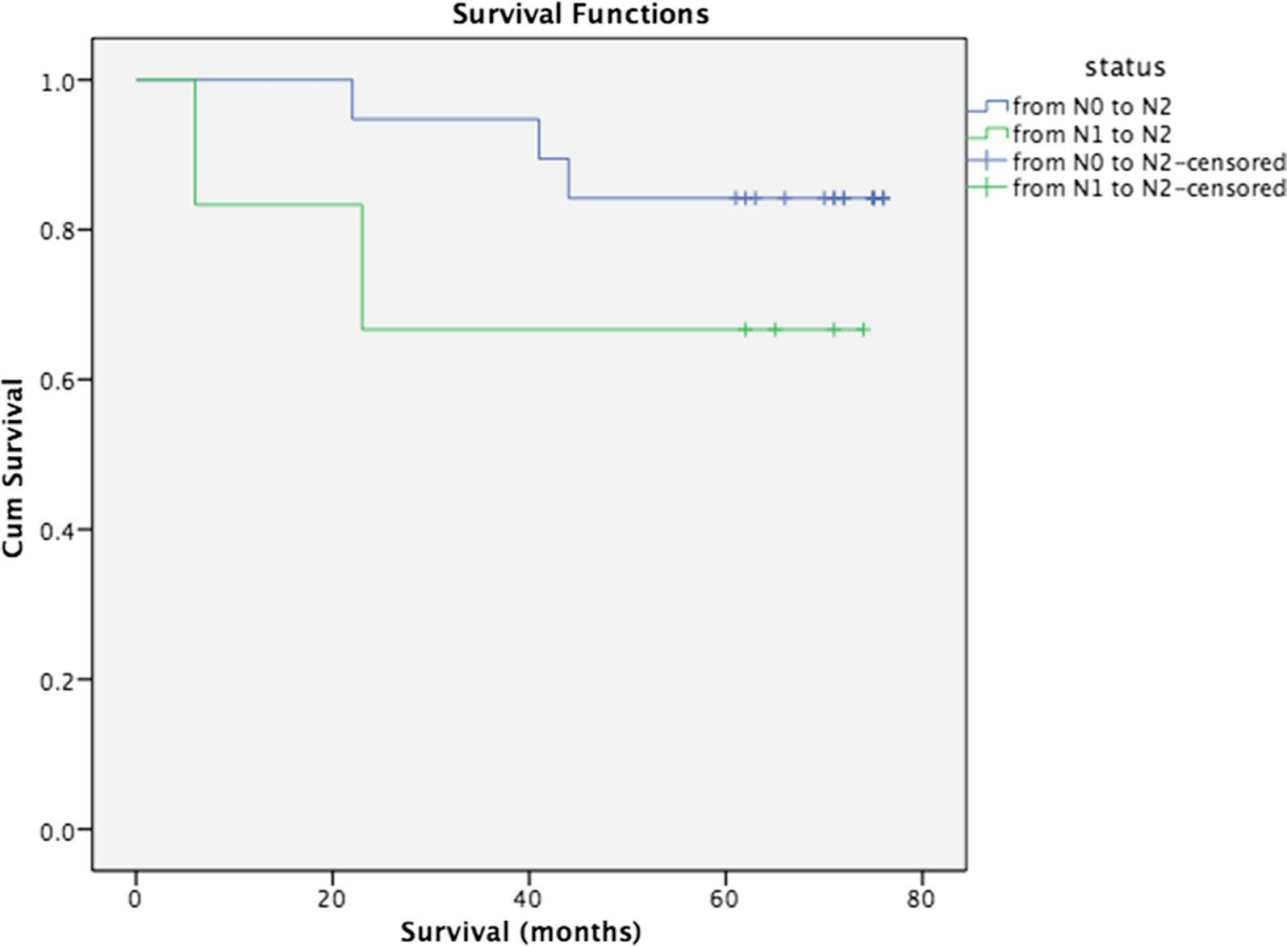
Survival analysis of upstaged patients from N0 to N2 and N1 to N2

We then performed survival analyses among the patients who upstaged to N2 disease. Upstaged group N1–N2 had worse survival compared to N0–N2 (*p*: 0.22) (Fig. 3). Even though it was not significant, worse survival could be the result of pathological N1 status, since it is known that pathological N0 has better survival than N1 [21, 22].

**Fig. 4:**
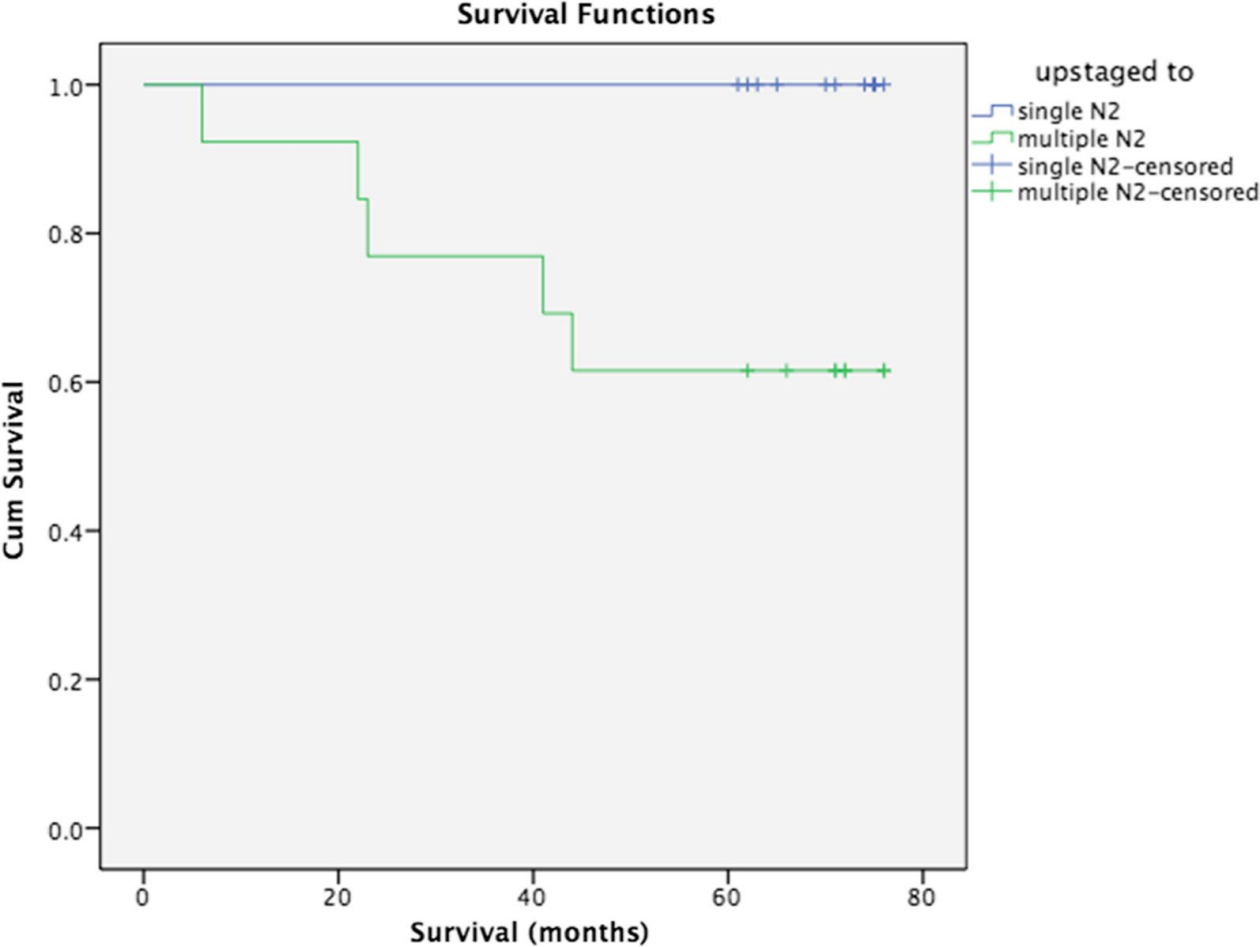
Survival analysis of upstaged patients to N2 with micrometastasis detected in a single MLN station or multiple MLN stations

Later we examined the impact of micrometastasis that is detected in more than one lymph node station in upstaged patients to N2 on the patients’ survival, as it is previously shown that multiple-station N2 has been correlated to worse survival [23]. We analysed patients who upstaged to N2 from either N0 or N1, we found that the multiple numbers of micrometastatic MLN from different stations have a significant impact on patients’ survival. Figure 4 shows that the patient who upstaged to N2 with a single-station MLN positivity tended to survive longer than the patient who upstaged to N2 with multiple-station N2 (*p* = 0.033).

Since another major aspect of survival of patients from NSCLC is a recurrence, as the last step, we analysed recurrences in our patient cohort. In total seven recurrences were observed six of them were in the upstaged group. We performed a chi-square test to see if multiple-station N2 have a similar impact on recurrence, it is found that recurrence was significantly higher in patients with micrometastasis in multiple-station MLNs than single-station MLNs (p-value 0.016).

## Discussion

Although the gold standard of treatment for early-stage NSCLC is primarily surgery, it is still suboptimal as the cancer recurrence after complete surgical tumour resection is 30% in Stage I and more than 60% in Stage IIB [24]. This suggests that potential occult micrometastasis might still exist after the surgery and it cannot be detected through postoperative diagnostic technologies. Occult micrometastasis may change the TNM staging of the tumour, thus, affecting prognosis. Even though micrometastasis is not included in the guidelines yet, postoperative detection of cancer cells in MLNs could help to predict recurrence and aid clinicians to decide on follow-up treatment strategies.

This study used the combination of EpCAM, CEACAM5 and CK19 as biomarkers to detect micrometastasis in MLNs. The lymph nodes were considered positive for micrometastasis when three biomarkers were overexpressed in tissues. We detected micrometastasis in 49 of 80 lymph nodes. We found that 86.67% were upstaged due to micrometastasis. In the previous MLN micrometastasis detection studies performed using the RT-PCR technique, the upstage rate of patients was shown to vary between 23.6 and 69%. Nosotti et al. [19] reported that micrometastasis was detected in 35 of 261 lymph nodes using the CEA as a biomarker, and at least nine patients were upstaged. Li et al. [25] stated that they detected micrometastasis in 36 out of 402 lymph nodes with MUC1 as a biomarker, and their upstage rates were calculated as 23.6%. Dai et al. [26] found the micrometastatic lymph node rate as 22% when they used fragile histidine triad diadenosine triphosphatase, FHIT, as a biomarker and 18% when they used cyclin-dependent kinase inhibitor 2A, CDKN2A, and the upstage rate was stated as 32.7%. Martin et al. [5] determined the upstage rates as 68.8% in their study using CEA as a biomarker. Even though we combined epithelial and tumour markers to increase specificity, our upstaged rate was out of this range of the previous studies. We chose these three markers, particularly because they are inclusive and expressed in NSCLC regardless of the subtype. The reason might be that the genes used in the previous publications were highly specific to a certain type of NSCLC and might not be expressed in all subtypes.

Although we detected micrometastasis in higher than the previous studies using a combination of markers is still more reliable than a single marker to detect micrometastasis. As to avoid bias in this study, we have re-evaluated all the remaining lymph nodes from our patient cohort. We found that one patient's micrometastatic lymph node was overlooked in the routine pathological examination previously and found CK19 positive during re-evaluation (Additional file 1: Fig. S1). Additionally, the presence of mesothelial cells was detected in micrometastatic lymph nodes during re-evaluation, which might have caused the high expression of the CK19 gene (Additional file 1: Fig. S2). However, in this study, we had only considered the lymph nodes as micrometastatic when all three markers' expressions were above the threshold. Therefore, the presence of mesothelial cells could be eliminated as EpCAM and CEACAM5 expressions are known to be absent in these cells. This incident showed that using three distinct markers increases the accuracy of the test while eliminating false positivity.

In the literature prognostic value of MLN micrometastasis is still controversial. In the study of Li et al., the 5-year survival rate of nodal micrometastatic patients was significantly lower than those without nodal micrometastasis (23.8% vs 44.1%; *p* < 0.05) [25]. In Dai et al. study, survival analysis showed that patients with nodal micrometastases significantly reduced disease-free survival and overall survival [26]. In the Martin et al. study, neither overall nor disease-free survival was associated with PCR positivity for nodal micrometastasis [5]. Since there is no uniformity to detecting micrometastasis in the guidelines the use of different biomarkers and including a small number of patients included in the studies may also be a factor for varying survival rates. In our study, the patients who have been upstaged tend to have slightly poorer survival compared to non-micrometastatic patients, but it failed to reach statistical significance. We also had a small study cohort and micrometastasis was detected in the majority of our patients, further evaluation will be required to determine larger cohorts to evaluate the effect of micrometastasis on patients’ survival.

MLN metastasis is known as one of the most significant prognostic factors in NSCLC. N2 disease reduces patients’ 5-year overall survival to 20–25% and it further decreases with an increasing number of N2 stations involved [23, 27]. According to proposals for the revision of the N descriptors in the 8th edition of the TNM classification, when there is a skip metastasis at N2 lymph nodes without N1 positivity (pN2a1), survival rates are higher when compared with both N1 and N2 positive disease (pN2a2) [21]. Even though micrometastasis's clinical importance is still debatable, there is a strong possibility that it could be an underlying factor of recurrence after curative resection. Indeed, we have shown that micrometastasis detected in patients in multiple MLN stations have a significantly higher recurrence rate compared to patients with micrometastasis detected in a single MLN station. Furthermore, we have observed that patients who upstaged to multiple-stationed N2 have significantly worse survival compared to single-stationed N2.

There are several factors impacting patients’ survival such as the tumour characteristics, the presence of comorbidities, the extent of the resection and the number of N2 stations involved yet it is known that the last factor is one of the most impactful on survival [2]. There are several studies published, and even though there are great heterogeneities among their treatment strategies, all stated that multi-station involvement proved to have a significant negative impact on survival [2].In this study, if the patients were staged according to the presence of micrometastases, they might have considered receiving additional treatments such as chemo and/or radiotherapy. Even though it still is not guaranteed that the patients who receive further treatment will live longer as chemotherapy and radiotherapy are effective treatment methods with various side effects, postoperative chemo- and radiotherapy for IIIA patients with positive lymph node micrometastasis shown to improve patients’ survival rate [28].

In summary, the prognostic value of MLN micrometastasis in NSCLC patients is still controversial. In this study, we have shown that micrometastasis might help to predict recurrence and survival in patients, particularly when it is found in multiple MLN stations. Further studies with larger cohorts are needed to confirm our findings which hold great potential decision-making process of treatment regimen postoperatively.

## Supplementary Information

**Additional file 1**: **Fig. S1**.Micrometastasis with a diameter of 303,94 um, which was overlooked on histological examination but detected by PCR examination., inset: Micrometastasis in high magnificationCK19 IHC staining of micrometastasis, inset: CK19 positivity in high magnification. **Fig. S2**.Mesothelial epithelial lining on the outside of the lymph node,CK19 IHC staining in mesothelial cells

## Data Availability

All data generated or analysed during this study are included in this published article.
