# Supplementary material for: Combination of CEACAM5, EpCAM and CK19 gene expressions in mediastinal lymph node micrometastasis is a prognostic factor for non-small cell lung cancer
Source: J Cardiothorac Surg. 2023 Jun 13;18:189. doi: 10.1186/s13019-023-02297-z (PMC10262366; doi:10.1186/s13019-023-02297-z)
Supplement: Supplementary file 1 — Additional file 1: Fig. S1.Micrometastasis with a diameter of 303,94 um, which was overlooked on histological examination but detected by PCR examination., inset: Micrometastasis in high magnificationCK19 IHC staining of micrometastasis, inset: CK19 positivity in high magnification. Fig. S2.Mesothelial epithelial lining on the outside of the lymph node,CK19 IHC staining in mesothelial cells [file 13019_2023_2297_MOESM1_ESM.pptx]

## Slide 1
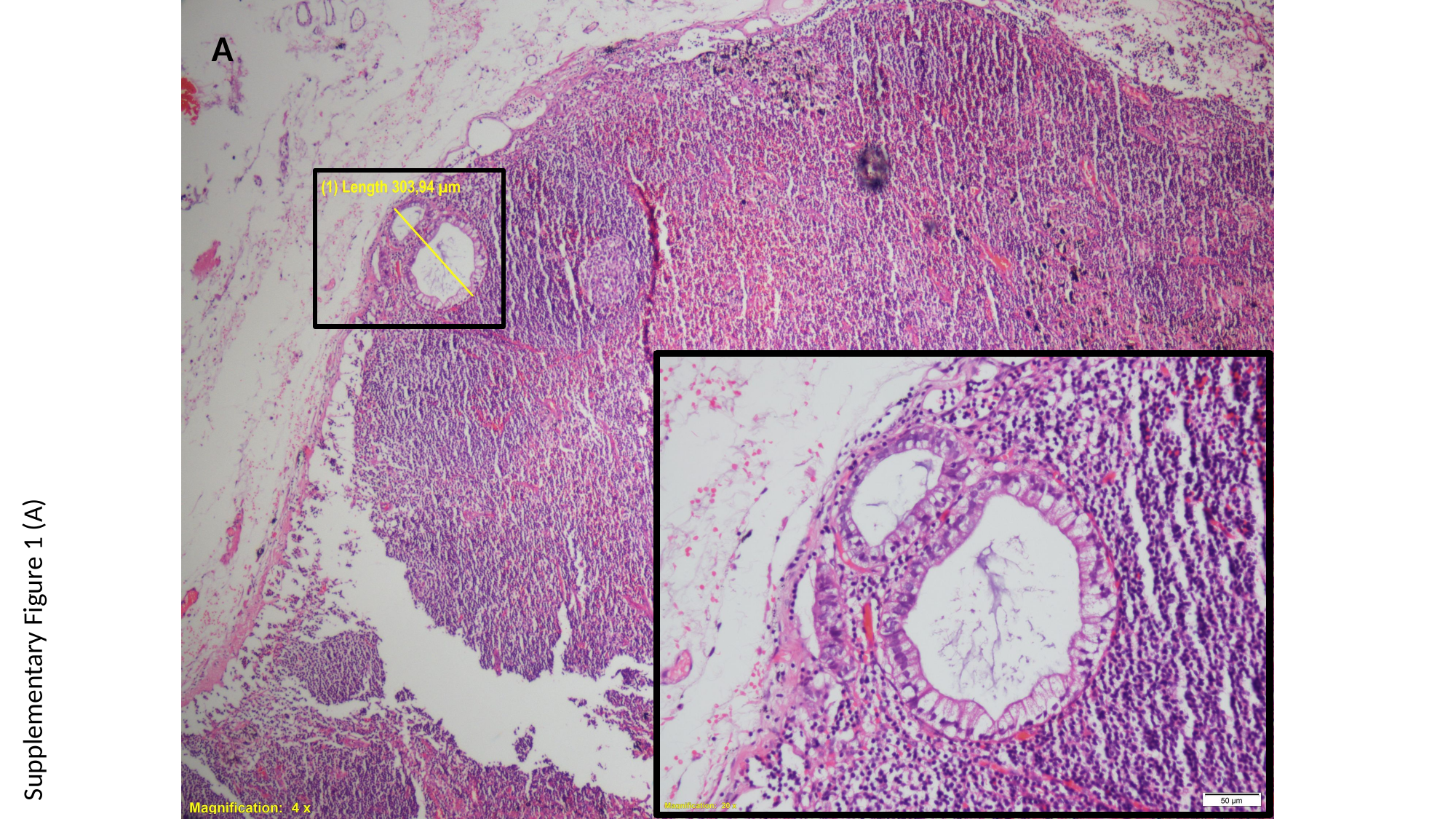

A
Supplementary Figure 1 (A)

## Slide 2
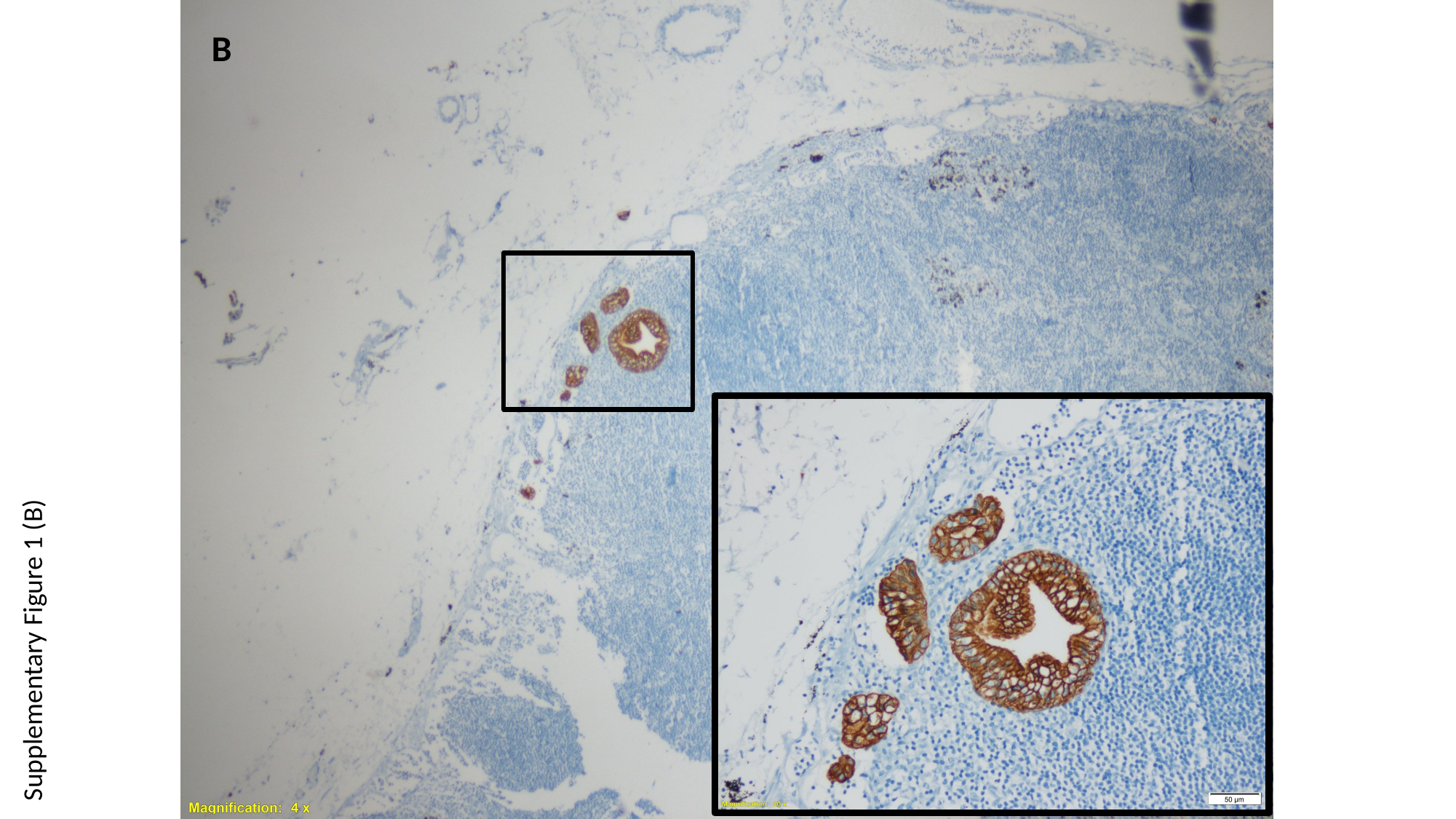

B
Supplementary Figure 1 (B)

## Slide 3
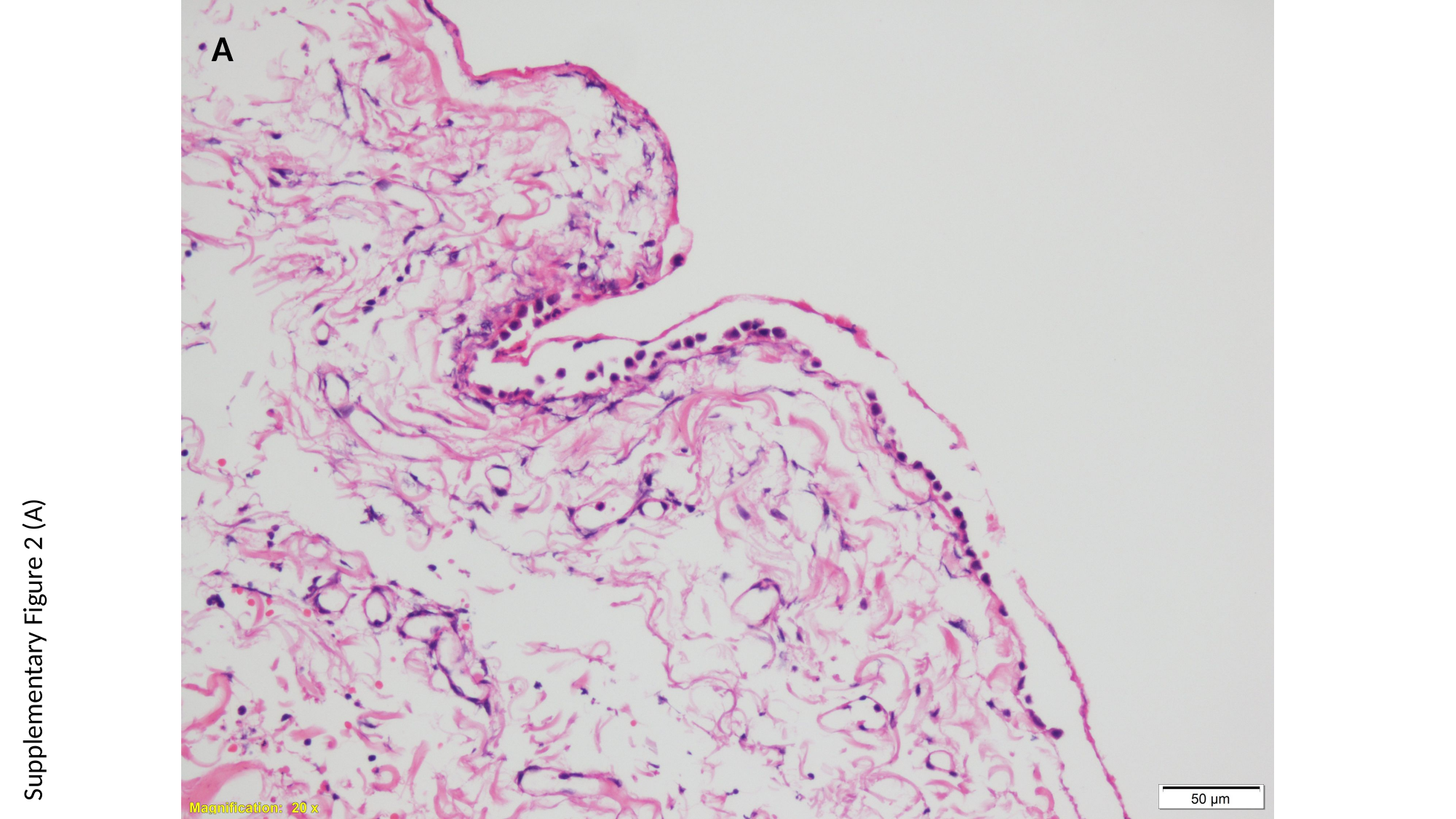

A
Supplementary Figure 2 (A)

## Slide 4
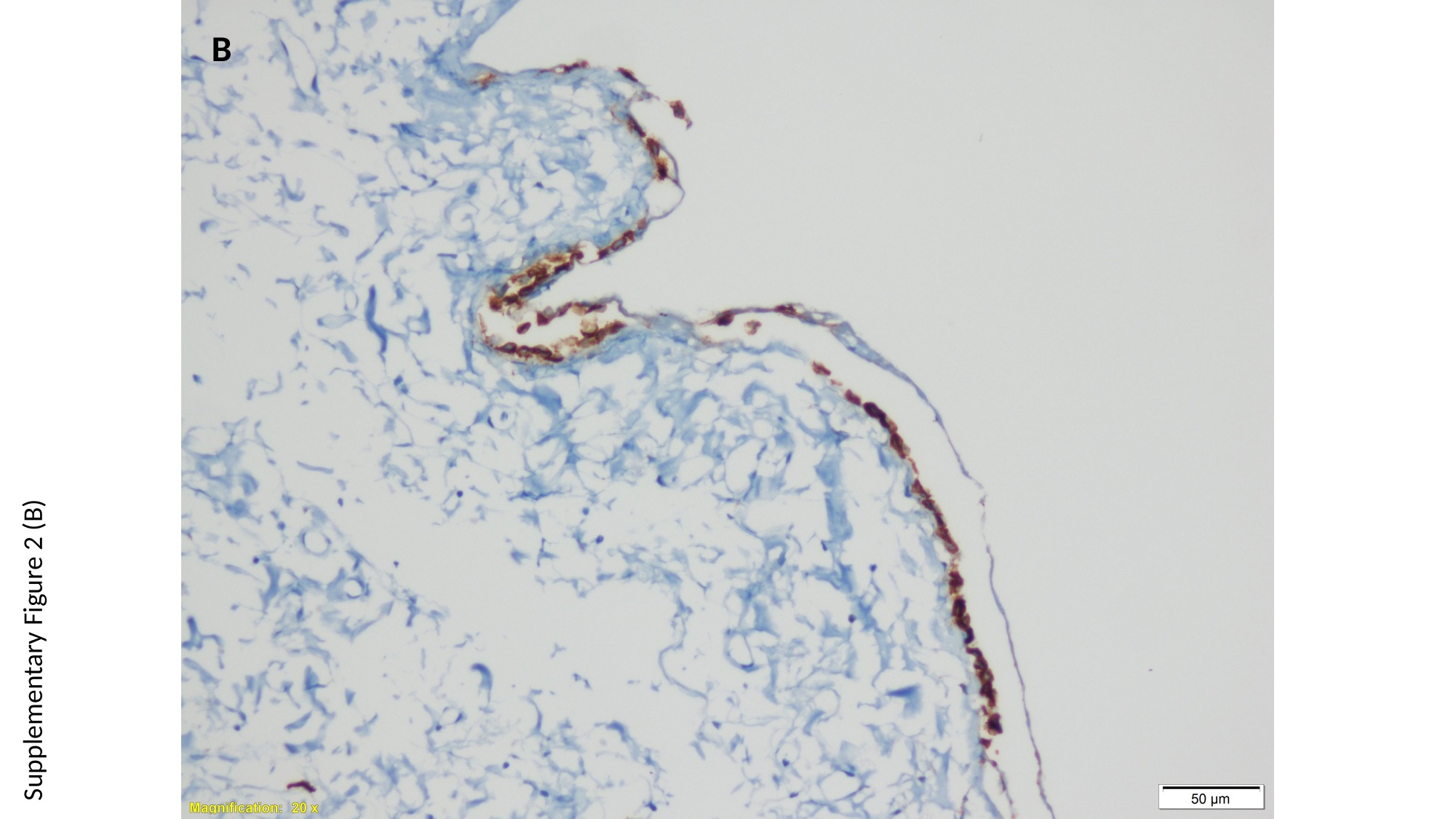

B
Supplementary Figure 2 (B)
